# An intra-neural microstimulation system for ultra-high field magnetic resonance imaging and magnetoencephalography

**DOI:** 10.1016/j.jneumeth.2017.07.016

**Published:** 2017-10-01

**Authors:** Paul M. Glover, Roger H. Watkins, George C. O’Neill, Rochelle Ackerley, Rosa Sanchez-Panchuelo, Francis McGlone, Matthew J. Brookes, Johan Wessberg, Susan T. Francis

**Affiliations:** a Sir Peter Mansfield Imaging Centre, University of Nottingham, Nottingham, NG7 2RD, UK; b Department of Physiology, University of Gothenburg, Gothenburg, 40530, Sweden; c Laboratoire de Neurosciences Intégratives et Adaptatives (UMR 7260), Aix-Marseille Université − CNRS, 13331 Marseille CEDEX 03, France; d School of Natural Sciences and Psychology, Liverpool John Moores University, Liverpool, L3 3AF, UK; e Institute of Psychology, Health & Society, Liverpool University, L3 5DA, UK

**Keywords:** Instrumentation, Stimulus generation, Low-noise amplifier, Nerve stimulation, Magnetoencephalography, Functional magnetic resonance imaging, Ultra-high magnetic field, Human, Microneurography, Tactile, Touch, Low-threshold mechanoreceptor

## Abstract

**Background:**

Intra-neural microstimulation (INMS) is a technique that allows the precise delivery of low-current electrical pulses into human peripheral nerves. Single unit INMS can be used to stimulate individual afferent nerve fibres during microneurography. Combining this with neuroimaging allows the unique monitoring of central nervous system activation in response to unitary, controlled tactile input, with functional magnetic resonance imaging (fMRI) providing exquisite spatial localisation of brain activity and magnetoencephalography (MEG) high temporal resolution.

**New method:**

INMS systems suitable for use within electrophysiology laboratories have been available for many years. We describe an INMS system specifically designed to provide compatibility with both ultra-high field (7 T) fMRI and MEG. Numerous technical and safety issues are addressed. The system is fully analogue, allowing for arbitrary frequency and amplitude INMS stimulation.

**Results:**

Unitary recordings obtained within both the MRI and MEG screened-room environments are comparable with those obtained in ‘clean’ electrophysiology recording environments. Single unit INMS (current <7 μA, 200 μs pulses) of individual mechanoreceptive afferents produces appropriate and robust responses during fMRI and MEG.

**Comparison with existing method(s):**

This custom-built MRI- and MEG-compatible stimulator overcomes issues with existing INMS approaches; it allows well-controlled switching between recording and stimulus mode, prevents electrical shocks because of long cable lengths, permits unlimited patterns of stimulation, and provides a system with improved work-flow and participant comfort.

**Conclusions:**

We demonstrate that the requirements for an INMS-integrated system, which can be used with both fMRI and MEG imaging systems, have been fully met.

## Highlights

• We propose an intra-neural microstimulation system for 7 T fMRI and MEG.
• This custom-built system removes issues with existing equipment.
• It provides efficient work-flow and improved participant comfort and safety.
• Stimulating single mechanoreceptors evokes activity in 7 T fMRI and MEG.
• Responses to unitary stimulation are shown for the first time in MEG.

## 1 Introduction

Intra-neural microstimulation (INMS) is a technique by which sensory nerve fibres can be stimulated electrically by delivering microamperes of current through an electrode inserted into a peripheral nerve during microneurography (Ochoa, 2010; Torebjörk et al., 1987). It is typically performed in conscious human participants to evoke synthetic percepts, and *single unit* INMS can be used to stimulate individual afferents (Ochoa and Torebjörk, 1983; Torebjörk and Ochoa, 1980; Vallbo, 1981; Vallbo et al., 1984). Here, a single mechanoreceptive afferent can be explored and characterised physiologically, and then stimulated electrically. Currents of ∼1–3 μA produce a clear tactile sensation (e.g. a vibration sensation from a fast-adapting type 1 (FAI) afferent and a pushing sensation from a slowly-adapting type 1 (SAI) afferent) and this synthetic *sensory projected field* corresponds well to the physiological receptive field (Vallbo et al., 1984). Since perceptually distinct, conscious sensations can be elicited from individual mechanoreceptive neurones in isolation (single unit), the contribution of the different mechanoreceptor classes to tactile sensation can be studied independently in a ‘quantal’ manner. It is possible to combine single unit INMS with neuroimaging to explore and contrast these quantal signals. The approach first involves microneurography, the recording of impulse traffic in a single primary afferent, resulting from mechanical stimuli applied to its receptive field. This is followed by INMS, to selectively activate the same afferent. A variety of electrical input patterns can then be used to probe subsequent central nervous system responses during neuroimaging.

Single unit INMS is in stark contrast to transcutaneous electrical stimulation of peripheral nerves, during which large numbers of different afferent types are non-selectively recruited, thus producing the sensation of paraesthesia (Burke, 1993; Johnson, 2007). Combining single unit INMS with neuroimaging allows the precise assessment of the brain’s response to tactile stimulation in a very controlled manner. We have previously used INMS to stimulate single units in conjunction with fMRI (Sanchez Panchuelo et al., 2016, Trulsson et al., 2001) and in combination with electroencephalography (EEG) (Kelly et al., 1997) to assess functional central nervous system responses. Sanchez Panchuelo et al. (2016) performed INMS during 7 T fMRI, but no study to date has progressed to using more complex or patterned stimuli, nor have they presented a sufficient sample size for separate analysis by receptor class.

The approach of INMS with neuroimaging presents several technical problems in terms of compatibility, the ability to collect sufficiently low noise recordings from neurones, and participant safety. In fMRI recordings, the switching of magnetic field gradients may be sufficient to generate currents in the long cables required to connect the participant to non-MR-compatible stimulation equipment (McGlone et al., 2002, Sanchez Panchuelo et al., 2016, Trulsson et al., 2001). Our previous research used a commercially-available stimulator system (AD Instruments, Castle Hill, Australia) for INMS in the MR scanner. However, since this system was not designed specifically for this purpose, it provided limited functionality in the both the level of stimulation precision which could be presented (steps of 1 μA, rather than the required 0.1 μA precision) and in the pulses which could be generated (only pre-programmed, simple pulse trains can be delivered). Additionally, manual switching between microneurography recording and INMS introduced problems, including long wait times when switching back to unit recordings (due to amplifier overload after stimulation), and additional preventative changes to the equipment were required to ensure that no extraneous currents were passed through the electrode to the participant (Sanchez Panchuelo et al., 2016). In EEG and MEG recordings, reed-relays employed to deliver stimulation in conventional INMS equipment, produce stimulation artefacts which limits their use (personal observations). To date, no study has demonstrated the use of MEG to study INMS induced responses.

Here, we designed an INMS system that would be compatible for use in a range of neuroimaging methods, specifically including 7 T fMRI and MEG. This system overcomes previous issues by allowing ease in switching between recording and stimulating, prevents extraneous electrical current discharge, and allows the delivery of customisable patterns of stimulation.

## 2 Methods

This section is divided into two parts. First, we describe the design specification of the INMS system, and follow this with the detailed design.

### 2.1 Design specification

The primary specification for an INMS system was that it should provide a safe connection to human participants whilst in use within the scanners. It must conform to, or exceed, specifications for an Internally Powered Medical Equipment, Applied Part (AP) classified, type BF (floating patient connection) as outlined in IEC 60101-1 (Medical electrical equipment − Part 1: General requirements for basic safety and essential performance). In addition, particular and collateral requirements for safe operation of Medical Equipment is pertinent to the design of the INMS system such as IEC 60101-2-33 (Magnetic Resonance Equipment), IEC 60101-2-26 (EEG) and IEC 60101-2-10 (Nerve and Muscle Stimulators). The INMS system was not required to be suitable for use with any other equipment requiring electrical connection, such as EMG, electrocardiography, or other stimulus devices.

The INMS system was designed to be capable of operating safely in the presence of high static magnetic fields (7 T MRI), large switched magnetic fields, and high radio frequency (RF) fields within the magnet bore. Conversely, the system required that INMS should have no detrimental effect on the quality of the image and functional data recorded by MRI and MEG scanners. For both MRI and MEG there was a requirement that there should be no ‘magnetic signature’, whilst related, this requires different design features for the two neuroimaging modalities. In addition, although the MEG environment imposes no particular safety conditions on the INMS system, the MR scanner has the potential for severe electrical interference effects, which should be addressed.

Considering MR safety, the INMS system was designed for use primarily within the Philips 7 T Achieva MR scanner (Best, Netherlands) equipped with a Nova Medical head volume transmit coil (Wilmington, MA). This set-up reduces the levels of RF present compared to a conventional 3 T MR scanner, as the head-stage amplifier unit is mounted on the forearm, which is not within the RF coil at 7 T, unlike a 3 T MR scanner which has a built-in body RF coil. The head-stage amplifier requires standard components with minimal ferrous content (i.e. using surface mount devices), so that there is no effect on scan quality. If those parts of the system in proximity to the bore are not considered magnetic when subjected to the standard deflection test, the INMS system can be designated as MR-compatible.

Connection of the amplifier to the patient via the electrodes creates a circuital loop, which can generate an electromotive force (EMF) in the presence of switched magnetic fields (audio or radio-frequencies). Low frequency magnetic fields are used in the imaging process should not induce voltages in the loop which disturb the stimulator, potentially producing false stimuli or shocks. RF (300 MHz at 7 T) could generate local heating due to antenna effects of the electrodes and leads. Local Specific Absorption Rate (SAR) levels in the limb should not be exceeded with the device connected. In addition there should be protection against local heating of the tissue round the electrode in the presence of high RF power.

For the MEG environment, there are no further participant- or equipment-related safety issues not already covered in the design for MRI compatibility. The INMS system was tested in a CTF 275 channel MEG scanner equipped with DSQ3500 acquisition electronics (Coquitlam, BC, Canada). In the MEG environment, there is a requirement for no participant movement, and care is taken to support the arm to ensure any is minimised. Therefore, any minimal ferrous content of the amplifier head-stage circuit board is not an issue for MEG recordings. It is desirable that the current loop generated by the INMS stimulus currents (<10 μA) entering the wrist through the electrodes does not generate a magnetic dipole of sufficient intensity to either be detected by the MEG system or cancelled by gradiometers and localisation methods.

There were two principal functional design objectives for the INMS system: (1) to perform microneurography, enabling placement of the electrodes in the median nerve and recording of unitary neuronal activity in the ‘Amplify’ mode; (2) to deliver a programmed sequence of pulse stimuli synchronous with the scanner acquisition when in the ‘Stimulate’ mode. In addition, a specific requirement was the ability to switch between these two modes of operation simply and quickly during mechanoreceptive afferent testing. It was essential that the switch-over should not produce any electrical shocks and could be controlled remotely by the operator even when the participant is within the scanner. Previous attempts at this experiment with commercial equipment required the use of mechanical switches, which in combination with large cable capacitances could cause a discharge when inside the nerve, producing an unpleasant sensation for the participant (personal observations). Furthermore, the amplifier in the commercial system was overloaded by the switching process, thus the experiment was delayed when switching back to ‘Amplify’ mode. The key functions of the equipment were that it should be controllable by the operator via a remote MR compatible box for ease and speed of operation during an experiment.

The amplifier head-stage was designed to have high impedance and low-noise when connected to the electrodes (having resistive and capacitive impedances of 100–500 kΩ). The nerve signals needed to be recordable and be fed back to the operator, both via an audio channel and visually on a screen. The system was designed to allow a basic level of spike analysis in order to assist in identifying afferent fibre types (e.g. fast- or slowly-adapting). The head-stage was designed to be small and light enough to mount on a body site near the electrode recording site, in order to make connections and cables to the electrodes as short as possible. The electronics were screened from RF and other electromagnetic interference, with the screen not causing any movement of the head-stage electronics due to induced currents caused by gradient switching, as movement could dislodge the electrode, resulting in the recording from the afferent being lost.

The stimulator was designed to be an analogue current amplifier in order to deliver a completely flexible pattern of stimuli pulses. The pulses were unipolar (switchable polarity), bipolar or D.C. balanced. The stimulus pulse was typically 200 μs duration with peak currents of up to 200 μA and 0.1 μA resolution. The maximum compliance was set to be of order 30 V in order to drive high impedance electrodes. In practice, less than 10 μA of current was used, with a voltage of the order of 1–2 V. The system was designed to monitor the current and voltage delivered to the electrodes for both test purposes and for impedance estimation.

### 2.2 Design in detail

**Fig. 1:**
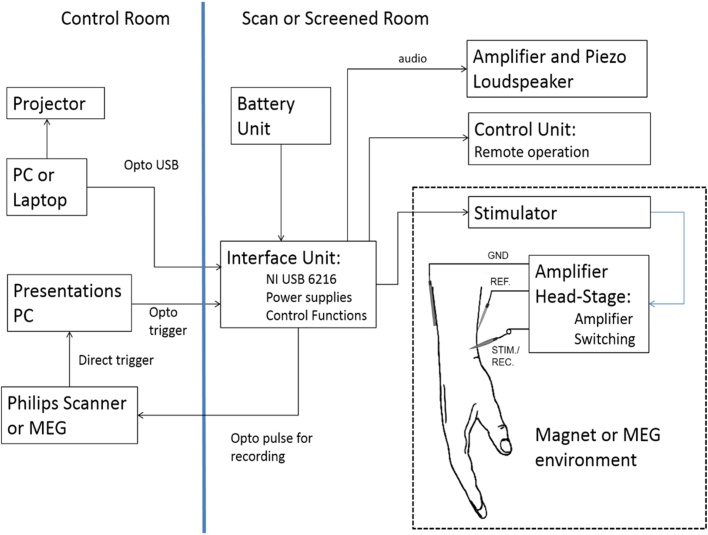
INMS system diagram. All equipment within the screened room is optically isolated for participant safety. Only the stimulator and head-stage units are approved for use within the bore of the 7 T magnet. The piezo loudspeaker and remote control unit are non-magnetic and can be used near the magnet during participant preparation. The interface unit and battery, whilst not projectile hazards, are not designed to work in high magnetic fields, and are situated at the edge of the screened room area. A similar set-up is employed within the MEG suite. The audio channel may, if preferred, be taken from the host control computer and played through speakers placed at the doors of the scan room.

Fig. 1 shows the functional blocks of the INMS system developed to achieve the design objectives. The first block comprises the computer controller devices: scanner computers (for MEG or fMRI); a computer to generate synchronisation pulses from the scanner and provide a trigger to the INMS system to initiate its stimulation sequence (using Presentation software; Neurobehavioral Systems, Berkeley, CA); and a host computer for the INMS control and signal recording. These devices are all positioned outside of the screened room of the MRI or MEG scanner, have a common ground, and are not isolated.

Optical fibres pass through to the scan rooms and all pieces of equipment are isolated from ground. These provide the participant isolation and all equipment inside the scan room runs off rechargeable batteries. The USB isolation is provided by a Corning (Hickory, NC) USB3.0 optical cable, which was modified to remove the cable “phantom” power supply to the isolated end, such that the power to the USB receiver electronics was replaced by a battery derived supply. This provided >10 GΩ isolation at a tested 500 V for all isolated units. The interface unit resided inside the MR screened room as far from the magnet as possible.

Within the interface unit a USB hub (TS-HUB3 K, Transcend Information Inc., Taipei, Taiwan) provides communications for a number of devices: a National Instruments (Austin, TX) USB 6216 provides all analogue and digital input and output functions; a PIC32 USB Audio interface (Microchip technology Inc., Chandler, AZ); and an Arduino Uno R3 (www.arduino.cc) dedicated to the control of the remote display. A 10 m cable runs from the interface box to the scanner bed (length defined by the layout of the MRI scan room), with an in-line stimulator unit 2 m before the unit containing the head-stage amplifier. As the stimulator unit contains no magnetic or electromechanical parts it can be placed near the MRI magnet or MEG. This location allows the reduction of the cable length between stimulator and electrodes to a minimum, and thus preserves pulse shape and minimises likelihood of any residual charge. Only the stimulator and head-stage units are defined as MRI-compatible within the bore of the magnet. The remote control box and piezo loudspeaker are nominally non-magnetic and can be used outside the bore of the magnet during microneurography. These are placed for the ease of use of the operator.

The key design objectives and novelty of this work are realised with the design of the amplifier and stimulator units. The success of the system relies on the ability to switch between recording and stimulation functions safely and quickly, without compromising the performance of either. The switching function relies on the adoption of opto-isolated FET analogue switches (H11F1 M, Fairchild Semiconductor Corp., Phoenix, AZ). These devices have a low on-resistance (200 Ω) and a high off-resistance ( > 100 MΩ), which result in a smooth transition between the two with the appropriately shaped LED current drive. This ensured any residual currents were dissipated slowly during switch over between functions. For safety, the current carrying capacity was limited to 500 μA, still well above the maximum value required for the INMS application.

**Fig. 2:**
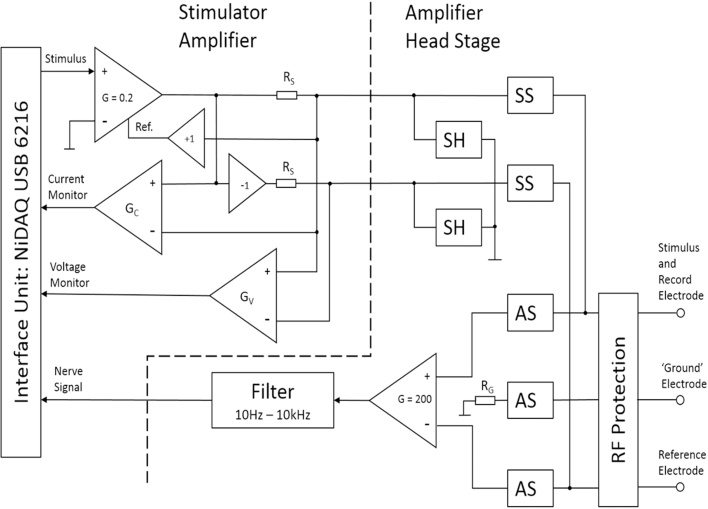
Amplifier and stimulator circuit elements showing switching. The amplifier switch (AS), stimulator switch (SS), and short switch (SH) are opto-coupled FET analogue switches. The head-stage, stimulator and interface blocks are shown in one diagram here for simplicity, but are in separate physical units, as described in Fig. 1.

Fig. 2 shows a simplified circuit diagram of the amplifier and stimulator units. The opto-FET switches are shown without their LED drives for simplicity. The switches were arranged in three groups: amplifier switch (AS) which connects the amplifier front-end to the electrodes; stimulator switch (SS) which connects the stimulator drive to the electrodes; and short switches (SH) which short circuit the stimulator. AS and SS switches are never on at the same time and the SH switches are on in amplify mode to reduce potential noise being injected into the front end. Note that the ‘ground’ electrode is only connected through R_G_ to the ‘ground’ of the electronics at time of amplification. This key feature ensures that return currents during stimulus drive all pass through the reference electrode, and thus allows a differential drive stimulus to be used. Hence, a 30 V compliance can be achieved with only 15 V power rails.

The front-end of the amplifier uses low-noise (6.5 nV/√Hz and 0.8 fA/√Hz at f = 1 kHz) JFET operational amplifiers (OPA2141, Texas Instruments, Dallas, TX) arranged as a differential amplifier. The inputs are D.C. coupled with an integrator feedback stage (time constant = 0.1 s) arranged to give a fast return to zero mean output. The gain of subsequent stages is arranged to give an overall head-stage amplifier gain of 200 with a bandwidth from 10 Hz to 10 kHz. The analogue output of the amplifier unit is sampled directly by the USB 6216 at a rate of 40 kHz. The power supply rails to the amplifier front-end are switched on only when the system is in amplify mode.

The stimulator electronics use standard operational amplifier devices which are capable of driving 200 μA outputs into a wide range of impedances. The driver amplifier employs a current sense resistor (R_S_) and positive feedback to the positive terminal of a differential amplifier to achieve a trans-conductance amplifier function. The drive potential across the electrodes is differential and this is achieved by an inverting voltage follower with a series impedance of R_S_. A useable bandwidth of 50 kHz into typical electrode impedances is obtained with the amplifier being unconditionally stable under all load conditions. The SH switch is used to test the current drive before connections are routed to the participant. The current and voltage applied to the electrodes are monitored and sampled for test purposes. Whilst it would be ideal to monitor these as close as possible to the electrodes they are monitored on the stimulator driver board. In INMS, the actual current used is determined by the threshold of induced sensation, and these measurements are only used as a guide so absolute precision is not required. The impedance of the electrodes is calculated and displayed as a guide to the operator as required. The power supply to the stimulator unit can be switched into high or low voltages depending on selection of high or low compliance modes, or off when not required. Switching the front-end circuits off when not required reduces the chance of instability or oscillations, plus saves battery lifetime. Power for the amplifier and stimulator units is derived from four 9 V 300 mAh PP3 NiMH rechargeable batteries arranged to give up to ±18 V supplies. These give at least two days intensive use of the INMS system.

To ensure compatibility with the MRI scanner, the amplifier and stimulator units have suitable screening and RF protection. 5 kΩ resistors are placed in-line with the electrode connections internal to the head-stage in order to reduce the magnitude of RF induced currents circulating through the electrodes, participant, and amplifier. In addition 100 pF RF traps are present on the connections to the board in order to prevent RF signals from affecting the operation of the stimulator. Hence, the 5 kΩ resistors and capacitors form a low-pass filter as seen by any RF pickup on the electrode wires. The additional capacitance does compromise the delivery of current to the electrode slightly but could be removed if MRI usage was not required. RF traps are used at the interface end of the cables to reduce the likelihood of common-mode RF signals passing down the cable. The internal shielding of the PVC amplifier and stimulator housings is achieved by using a copper coated non-woven polymer fabric. This gives good RF performance but has a relatively low conductance at low-frequencies. Eddy currents induced by magnetic field switching or movements are substantially reduced compared to use of copper film screens. In addition the ground plane of the amplifier board is carefully designed to minimise eddy current effects, which could lead to vibrations.

**Fig. 3:**
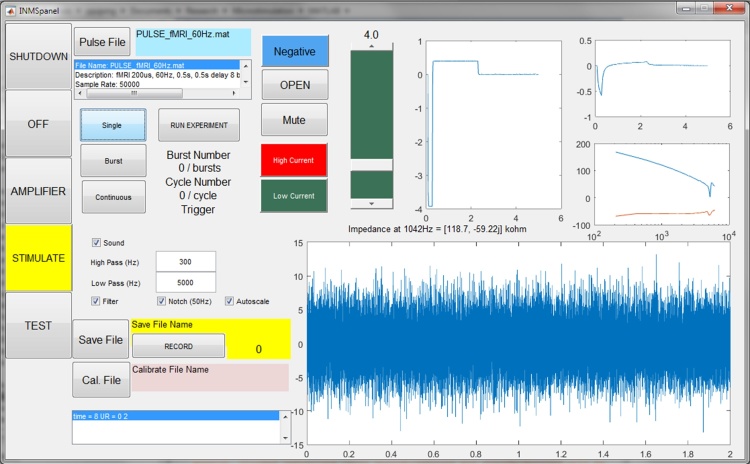
Software front panel. Function control buttons are on the left. Stimulus control and functions are at the top, and amplifier functions are in the lower half of the panel. A scrolling display shows the latest 2 s of acquired signal which can be directed to the audio channel and/or buffered to a recording file. The data shown illustrates low-noise recordings from typical electrodes in saline within the bottle phantom. In this graphic the display is set to auto-scale whereas the scale is usually fixed to ±50 μV during experiments. A slider controls the set peak current value which can be updated from the computer or controlled by a rotary encoder on the remote control unit. Other functions and panels are described in the text.

All devices are connected through the isolated USB and are controlled by software custom-written in MATLAB 2015a (The Mathworks, Natick, MA), specifically using the Data Acquisition Toolbox for control of the 6216 NiDAQ. A graphical user interface based front-end (Fig. 3) allows control of all functions, automatically ensuring the correct sequence of power supply and opto-switch switching. Acquisition data is sampled at 40 kHz, filtered within a selected band (usually 300–5000 Hz), recorded (if required), buffered to a rolling chart display, and streamed to the host computer’s audio device. The latter is useful, as the operator can then route the audio feed to either the PC’s own internal audio, external speakers or back through the USB connection to the PIC32 audio driver in the interface unit and onwards to piezo speaker (or operator earpiece) as required.

A spike analysis module can be built into the acquisition data stream if required or applied to recorded data retrospectively. The PC used (L540 Lenovo, China) takes 10 ms to acquire, filter, record and display the equivalent of 50 ms of data, so any further analysis should fit easily within the remaining time-frame.

In ‘Stimulate’ mode power is applied to the stimulator driver with the electrode amplifier powered down. The opto-FETs SS are closed with SH and AS switches left open. Hence stimulus current is routed to connect the driver to the electrodes. Software-generated pulse sequences are produced at 50 kHz sampling rate, from a data file which defines the overall protocol. There is complete flexibility in the waveforms generated and this opens the possibility of applying any pattern of INMS (e.g. one that mimics the natural firing of mechanoreceptive afferents). The remote-control box allows the operator to control the important functions of the software, such as the Amplify/Stimulate switching function, controlling applied current, and initiating bursts of pulses for determining the current threshold for a perceived sensation. To assist the operator, a visual display of the screen of the INMS control computer is projected into the scan room (a standard feature of most MEG and fMRI laboratories), as well as an alphanumeric display on the remote control box giving information including the electrical current level and impedance. The latter display is achieved using the SPI port of the Arduino board. Software is written for the Arduino to interpret a string of text sent to an attached COM port from MATLAB, and to put it on the screen.

Two 15 mm tungsten electrodes (one insulated and one uninsulated) (FHC, Bowdoin, ME, USA) were inserted through and glued to the cap of a polythene bottle (200 ml, 50 mm diameter). A silver plated ‘ground’ electrode wire was also attached to the cap. The bottle was filled with saline (0.5% by weight NaCl) such that the electrodes were covered. Leads were attached in order to plug into the amplifier head-stage. This bottle phantom was used for amplifier noise and stimulator tests prior to experiments taking place. In addition the ‘ground’ electrode was connected to copper tape contacts on the outside of the bottle. Hence for test purposes only an operator could hold the bottle phantom as a reliable test for electromagnetic interference in the scanner environments. In addition, these test scans also allowed the assessment of signal-to-noise measures when inside and outside of the scanner.

## 3 Experimental

Ethics for experiments on human participants using this equipment and associated imaging protocols was granted by The University of Nottingham Medical School Ethics Committee (E09022012, Experimental Protocol, Participant Information Sheet, informed Consent Form and MRI safety questionnaire). All participants were given detailed information about the procedure and signed a written consent form. All procedures were conducted in line with the Declaration of Helsinki.

**Fig. 4:**
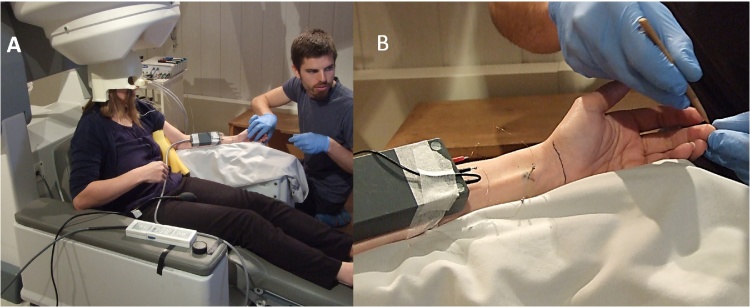
Detection of units in MEG. (A) The subject is seated in the chair slightly lowered below the normal MEG scan position. This is for their comfort during set-up of the electrodes and characterisation of the units found. (B) The amplifier head-stage is shown taped to the forearm with the electrodes visible. The upper electrode (red terminal) is the median nerve electrode with the reference (current return) underneath placed subcutaneously. The black wire is attached to the unseen ‘ground’ electrode. The stimulator box is not shown in these photographs but is positioned on the floor close to the subject’s feet. The operator is locating the afferent by stroking or touching the skin with a monofilament whilst listening to the audio and observing the traces, as shown in Fig. 5. The subject is wearing their individual head-cast. The wires into the head cast are actively driven location markers for head registration. For fMRI the cables going to the amplifier box are routed such that they cannot touch the subject and create an RF antenna loop which couples to the body.

Four subjects participated in sessions involving characterisation of a single mechanoreceptive unit followed by INMS of this unit during either 7 T fMRI or MEG acquisition. For the fMRI experiment, the participant lay on the scanner bed outside the bore of the magnet, while for the MEG experiment the participant was seated just below the level of the sensor helmet and wore a custom-fitted head-cast (Chalk Studios, London, UK) to prevent head movement (Liuzzi et al., 2016, Meyer et al., 2017), as shown in Fig. 4. In our MEG system, there was an interference signal conducted through the MEG earth which does elevate the noise floor as measured by the INMS. By setting up the subject with their head removed from the MEG helmet and switching off the power to the bed and gantry controller, this noise was reduced to an acceptable level.

An insulated tungsten electrode (FHC, Bowdoin, ME; length: 15 mm) was inserted percutaneously into the median nerve, approximately 3 cm proximal from the wrist fold, to stimulate and record from single mechanoreceptive afferent units. A similar, un-insulated electrode was inserted just under the skin 4 cm away. Once a single unit recording was identified in amplifier mode, it was characterised based upon its response characteristics to stimulation with a monofilament as fast-adapting type 1 (FAI) or type 2 (FAII), or as slowly-adapting type 1 (SAI) or type 2 (SAII) (Vallbo and Johansson, 1984). Unitary data were band pass filtered (0.3-4.5 kHz) for visualisation online and recorded, using custom software written in MATLAB. Nerve data were only registered when the subject was positioned outside the bore of the 7T scanner or just below the sensor helmet in the MEG. Offline, the data were processed with a band pass smoothing filter in MATLAB (0.16-2.5 kHz) to remove high frequency artefacts from MEG recordings, and exported to Spike 2 software (CED, Cambridge, UK) for identification of spikes based on a combination of amplitude and spike shape. The INMS system was then switched to ‘Stimulate’ mode and positive current pulse trains of 60 Hz for 1 s were manually delivered by the operator, incrementing the current until a sensation was reported by the participant, or until 8 μA. If the perceived location of the electrically elicited sensation exactly matched the location where mechanical stimulation of the skin generated a response (Torebjörk et al., 1987), the stimulation protocol was performed. At this point the participant was moved either into the bore of the MR scanner, or into the MEG sensor helmet. After moving the participant, INMS was re-evaluated to ensure that the perceptual response had not changed or was lost, and the stimulation current was adjusted if necessary.

7 T fMRI scanning protocols and INMS pulse definitions are identical to those reported in Sanchez Panchuelo et al. (2016), except from the spatial resolution which was here increased to 1.25 mm isotropic. A burst of 60 Hz 200 μs current pulses of 1 s (0.5 s on and 0.5 s off) were repeated 8 times in a sequence, followed by a rest period of 23 s. This was repeated for a total of 8 cycles. The MEG protocol consisted of a 1 s burst of 60 Hz 200 μs current pulses followed by a 10–10.5 (a randomly selected delay period) second rest period, repeated for 80 cycles in blocks of 10 cycles. MEG data were recorded at 1200 Hz using a 275 channel MEG system (CTF, Coquitlam, BC), with the reference array set to a synthetic 3rd order configuration.

After completing an fMRI stimulation cycle, or one block of MEG stimulation, the sensation was checked by asking the participant if the sensation had changed in intensity or quality. If the sensation had faded during stimulation due to minute dislodgements of the electrode, the stimulation current was adjusted to give a comparable intensity of stimulation, with the same quality (Torebjörk et al., 1987).

fMRI image data were analysed using a General Linear Model in mrTools (http://www.cns.nyu.edu/heegerlab). Statistical maps were formed by thresholding (Z > 3.08) after false discovery rate correction and projected onto a flattened representation of the contralateral central sulcus to compare the spatial localisation with previously acquired finger somatotopy in primary somatosensory cortex (for more details, see (Sanchez Panchuelo et al., 2016)).

MEG data were visually inspected for artefacts, such as SQUID resets, and magnetomyographic or magnetooculargraphic contamination, where trials containing excessive artefacts were removed. To allow for the reconstruction of sensor data at the source level, three head position indicators (placed on the nasion, and left and right pre-auricular regions of the participant’s face) were periodically energised to locate the participant’s head within the dome. The coils’ positions relative to the brain were determined during the manufacture of the head casts. Data were frequency filtered into the beta band (13–30 Hz) and reconstructed in source space using a beamformer (Robinson and Vrba, 1999; Van Veen et al., 1997), lead fields were computed using a dipole approximation (Sarvas, 1987) in conjunction with a multiple local sphere head model (Huang et al., 1999). Dipole orientation was determined using a non-linear search for optimum signal to noise ratio (SNR). To locate the source, a pseudo-T-statistical image was generated across a 2 mm isotopic grid spanning the right hemisphere’s pre- and post-central gyri (active window: 0–1 s after stimulus onset; control window: 8–9 s after stimulus onset). After determining the source location by finding the maximum absolute pseudo-T score, 1–150 Hz filtered sensor data were source reconstructed at the target location and a time-frequency spectrogram (TFS) generated.

## 4 Results

A number of experiments and measurements were made to ensure and demonstrate the safe working of the equipment before use with humans. The results in detail, methods and discussion can be found in the Data in Brief document accompanying this paper. In summary, during fMRI sequences, no temperature rise local to the electrodes due to SAR heating effects was discerned (±0.1 K). In addition, measurements showed a level of current injection due to magnetic field gradient switching was less than 10 nA, well below the level which would influence applied stimulation patterns or cause pain.

Test recordings from electrodes with known impedances demonstrated an acceptable low-level of noise, with no undue noise attributable to the extra series impedances of the switches and RF protection. A low-noise recording from the bottle phantom is shown in Fig. 3. This noise recording has an RMS amplitude of 3.3 μV, equivalent to 48 nV/√Hz, which is in-line with an expected value of 41 nV/√Hz for a 100 kΩ resistor at room temperature. The latter value assumes an ideal filter and a frequency invariant source impedance − neither of which are true for this type of electrode. However, similar baseline noise levels are observed in practice.

Fig. 3 shows a 4 μA current pulse being delivered to the bottle phantom electrodes, which is of negative polarity with respect to the reference electrode. The pulse has an equalisation reverse polarity of 1/10 peak amplitude with 10 times duration. The associated electrode voltage (top right) and impedance as a function of frequency (middle right graph) can be seen, with the measured impedance at 1 kHz (printed as 118.7, −59.22 j kΩ). Periodic measurement of electrode impedance gives the operator an insight into the condition of the electrode.

**Fig. 5:**
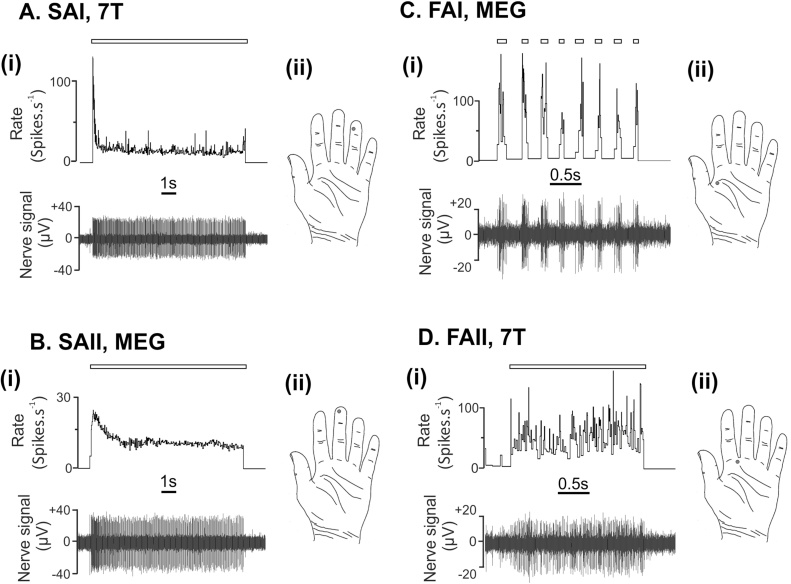
Examples of recordings from individual mechanoreceptive afferents in the 7 T fMRI and MEG environments. Recordings from individual SAI (A) and FAII (D) mechanoreceptive afferents in the 7 T fMRI environment. The SAI recording shows the response to long-lasting indentation with a 2 g monofilament and the FAII recording shows the response to lightly blowing onto its receptive field. The recordings are made while the participant is lying on the 7 T scanner bed, with the head-stage positioned on the participant’s arm at the bore-end of the scanner. The magnetic field experienced by the amplifier head-stage is ∼ 0.5 T. (B) Recordings from individual FAI (C) and SAI (B) mechanoreceptive afferents in the MEG environment. The FAI recording shows its response to repeatedly moving a wooden stick across its receptive field and the SAII shows its response to a sustained indentation with a blunt wooden stick. The MEG recordings are made with the participant positioned just outside of the helmet in the MEG scanner. In all recordings, the bar above the trace indicates the timing of the respective receptive field stimulation. In both environments, amplifier noise was less than 5 μV RMS with a 300–5000 Hz filter bandwidth. Note the higher noise floor in the MEG recordings.

Fig. 4 shows the process of setting up the subject in the MEG system. The amplifier head-stage and electrode arrangement can be seen. The operator is finding the nerve signals at this point by lightly touching the hand and fingers. When a unit has been found it can be characterised by its response to touch or pressure. The setup phase for fMRI is similar except the subject is lying down and no head-cast is used. Fig. 5 shows example recordings from both the 7 T fMRI (Fig. 5A and 5D) and MEG (Fig. 5 B and 5C) environments. Physiological single unit recordings are shown from each type of mechanoreceptive afferent found in the glabrous hand skin. Hence, this demonstrates that it is possible to obtain clear recordings from all mechanoreceptor types in both MRI and MEG environments. The recordings have a low noise-floor, with very little external interference and easily distinguishable neuronal spikes.

**Fig. 6:**
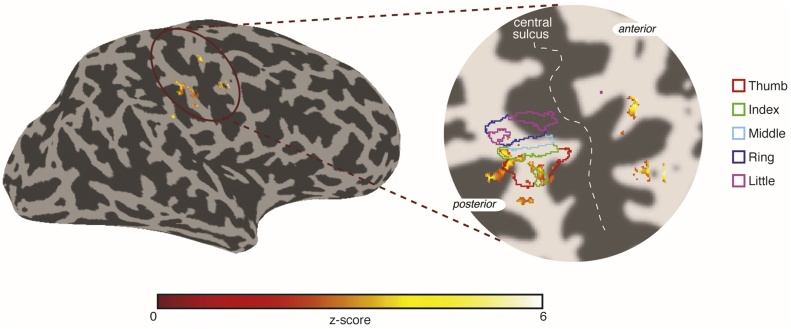
Example 7 T fMRI activation in response to INMS stimulation (60 Hz) of an FAI unit located on the base of the index finger. Data is shown on an inflated brain and activity can be seen to localise within contralateral S1 (note that the activation posterior of the thumb region −red outline- is due to a large draining vein). On the expanded map the INMS-induced activity is shown in detail along with coloured lines indicating the borders of each finger representation obtained from a previous travelling wave somatotopy experiment in the same participant.

Fig. 6 shows an example of fMRI activation from the stimulation of a single FAI unit at the base of the index finger, using 60 Hz pulses. The fMRI data show statistical activation maps (Z > 3.08, FDR-corrected) to INMS of the unit overlaid onto a surface reconstruction of the contralateral (right) hemisphere and on a flattened patch of the central sulcus. INMS activation patterns in contralateral primary somatosensory cortex (S1) area are consistent with the expected spatial localisation from finger somatotopy derived from a travelling wave vibrotactile paradigm (Sanchez-Panchuelo et al., 2012, Sanchez Panchuelo et al., 2016). No difference in image SNR was demonstrated on phantom data, and no significant difference in the temporal signal-to-noise ratio (tSNR) of the fMRI data was found between the INMS stimulation ON period (tSNR grey matter: 46 ± 4) and OFF period (tSNR grey matter: 44 ± 5) or tSNR with no INMS system in place (tSNR grey matter: 43 ± 4), demonstrating that the INMS system does not alter MR image quality.

**Fig. 7:**
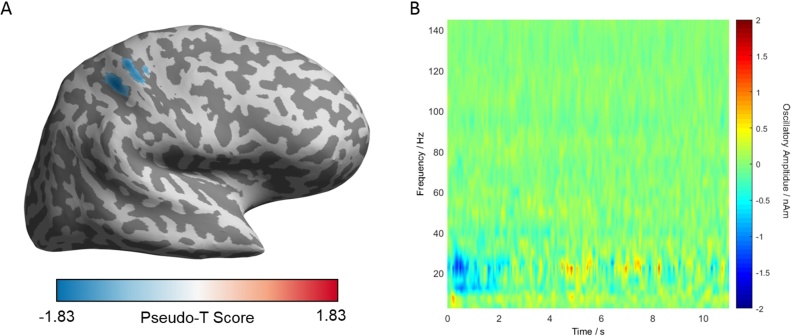
Results from a single INMS experiment in MEG, in which stimulation of an SAI unit located on palm of the hand occurred. (A) The localisation of task-induced changes in power, projected onto an inflated cortical surface, showing the location of the source to be within the dorsolateral postcentral gyrus. The image is threshold at 80% of the maximum value for clarity. (B) Trial-averaged time frequency spectrogram (TFS) of a virtual electrode placed at the source location, derived from the maximum absolute pseudo-T score.

Fig. 7A demonstrates localisation of the neuronal activity for single unit INMS of an SAI afferent in MEG located in the palm of the hand. Here, to aid visualisation of the source location, the pseudo-T statistical image has been projected onto an inflated cortical surface of the participant using the Freesurfer analysis suite (http://freesurfer.net). The source image shows that the maximal change in beta power during the stimulation was located within the dorsolateral postcentral gyrus. The time-frequency spectrum plot in Fig. 7B shows the average change in spectral power across the 80 trials. Within the 15–30 Hz band, the characteristic event-related cortical desynchronisation during the time of the stimulus (0–1 s) and resynchronisation after cessation of the INMS is seen. Note, at 60 Hz no clear power modulation specific to the trial is seen, suggesting that with the applications of appropriate environmental noise cancellation and spatial filtering methods, artefacts from passing current from the INMS system into the nerve are negligible.

## 5 Discussion

These results demonstrate the superior level of measurement using this specially-designed INMS system, and show that its performance is comparable to equipment used in a dedicated microneurography laboratory. Further, the system provides overall ease of use in setting up, switching of function, and workflow, meeting all of the requirements in terms of safety and usage. Specifically, low-noise recordings from individual mechanoreceptive afferents were recorded in both the 7 T MR and MEG scanners. This made it possible to search for, identify, and record from single units, which were then subject to INMS. The system allowed precise electrical pulses to be sent back down the electrode, to excite a single afferent and produce a quantal sensation (cf. Torebjörk et al., 1987; Vallbo et al., 1984). This clear perceived sensation continued to be artificially-induced on re-stimulation once the participant had entered the 7 T magnet bore or MEG sensor helmet, allowing the combination of single unit INMS with concurrent neuroimaging.

From a safety point of view, no incidental electrical micro-shocks were produced (cf. the previous commercially-available system used in Sanchez Panchuelo et al. (2016)) and the present system provides complete user control, with a dedicated control box near the microneurographer, as well as full computer control outside the scanner. Our results show the potential in combining single unit INMS with 7 T fMRI and MEG. Although not shown here, it is likely that such a system can be combined with other neuroimaging methods, such as EEG, electrocorticography, and functional near-infrared spectroscopy, as well as other neurophysiological monitoring (e.g. electrocardiography, eye movements).

The present system can be used to perform INMS during neuroimaging, which greatly facilitates the study of, primarily cortical, responses to controlled input from somatosensory afferent nerves. We provide the first demonstration of such an application in MEG. This system will allow the contribution of different afferent classes to the processing of somatosensory information throughout the brain to be evaluated. Using high spatial resolution 7 T fMRI, it is hoped that the activity within- and between cortical layers in primary somatosensory cortex can be visualised. Similarly, using high temporal resolution MEG, the precise cortical dynamics over time in response to INMS of different afferent types can be assessed. The compatibility of the system with multiple neuroimaging techniques will potentially allow for the combination of data across methodologies, to further elucidate the spatio-temporal dynamics of cortical somatosensory responses. Results from separate experiments in the same subject in MEG and 7T fMRI, may be combined to aid in the understanding of the relationship between locations of the neurovascular response (fMRI) and MEG inverse localisation. Using the precise signal generated during single unit INMS should aid in elucidating localisation accuracy between techniques, as well as understanding detailed cortical signals. Presumed compatibility with additional neuroimaging techniques will allow simultaneous acquisition of neuroimaging data such as EEG/fMRI, EEG/MEG, or EEG/functional near infra-red spectroscopy. Furthermore, the INMS system allows unconstrained electrical patterns to be delivered; hence a variety of different frequencies can be tested, with the possibility of delivering other more variable patterns, such as those derived from the natural firing of mechanoreceptors. These studies will aid in understanding the fundamental workings and connectivity in somatosensory circuits.

## 6 Conclusions

We show the implementation and application of a dedicated system for single unit INMS of mechanoreceptive afferents, during combined 7 T fMRI or combined MEG acquisition. This is the first demonstration of the feasibility of performing single unit INMS measures while recording MEG signals. The INMS system goes beyond previous systems, where it provides safe and effective operation, and provides comparable neuronal recording and stimulation to equivalent procedures in dedicated microneurography laboratories. Using this system will enable the smooth and efficient collection of high spatial and temporal resolution data from single unit INMS during neuroimaging. Due to the unconstrained stimulation capabilities of the system, further experiments will probe a variety of artificial input INMS patterns and examine the detailed and precise central responses generated.
