# Supplementary material for: An intra-neural microstimulation system for ultra-high field magnetic resonance imaging and magnetoencephalography
Source: J Neurosci Methods. 2017 Oct 1;290:69–78. doi: 10.1016/j.jneumeth.2017.07.016 (PMC5594527; doi:10.1016/j.jneumeth.2017.07.016)
Supplement: Supplementary file 1 [file mmc1.docx]

An intra-neural microstimulation system for ultra-high field magnetic resonance imaging and magnetoencephalography

# Supplementary Information (Data in Brief)

## Glover et al

# Safety within the 7T MRI scanner

There are two potential sources of electromagnetic (EM) interaction occurring within the MRI scanner environment which could potentially cause harm to the subject. These are: low frequency (100 Hz – 10 kHz) magnetic field gradient switching (30 mT/m switched in 200 µs) used for spatial encoding; and radio-frequency (RF) induced currents in the tissues and electrodes caused by high power (>200 W) RF excitation pulses. The former may induce electric currents which cause nerve stimulation in the body known as Peripheral Nerve Stimulation (PNS)([1](#_ENREF_1)). The latter have the potential to cause local heating of the tissues.

## Low frequency EM interactions

It is instructive to consider the likelihood of induced voltages having either an effect on the experiment itself, or as a hazard. If we assume that the loop made by the wires from the head-stage to the electrodes and through the wrist is a circle of diameter of 50 mm, then the induced EMF can be calculated from this area and the rate of change of magnetic field. As the wrist of the subject is outside the linear region of the gradient coils, the maximum rate of change of magnetic field can be estimated to be of order 75 T/s, giving a corresponding worst case maximum EMF of 150 mV. As this is approximately 1/10 of the peak pulse voltage required to excite a single unit, it will not cause stimulation. In practice, the INMS system has been designed to allow the voltages and currents flowing to be estimated during ‘Stimulate Mode’ and monitored and recorded if required. Figure S.1 shows a short excerpt from such a recording using the bottle test-phantom described in the main Methods section. This shows that the loop picks up a maximum of 35 mV peak during the fat suppression part of the multiband fMRI sequence and generally lower at approximately 20 mV during the EPI readout. These values are much lower than the theoretical assumption above. As the current driver is enabled during recording then the feedback mechanism copes well with the induced voltage, maintaining the current to within 10 nA of zero (i.e. the voltage measured exactly balances the induced voltage resulting in zero net EMF around the whole loop). It is also worthy of note that as there are RF pulses present during this recorded excerpt then if the current driver was affected by the RF pulse then there would be evidence of a current disturbance. However, this effect is not significant. If the INMS system is in either the ‘Off’ or ‘Amplify Mode’, then only very low current can flow through the electrodes as there is effectively a break in the loop and the impedance of the loop and electrodes is greater than 100 kΩ.

The possibility of low-frequency heating of the tissue around the electrodes is extremely low, since only a maximum possible power of the order of nW (worst case) could be dissipated during these measurements even in the case of the electrode wires becoming shorted together.

A simple but persuasive argument against the likelihood of PNS would be that the INMS experiment itself is the most sensitive test of potential PNS, having placed the electrode within the nerve sheath itself. No subject has reported any sensation un-related to the deliberate controlled stimulus process during either the fMRI or preparatory scans.


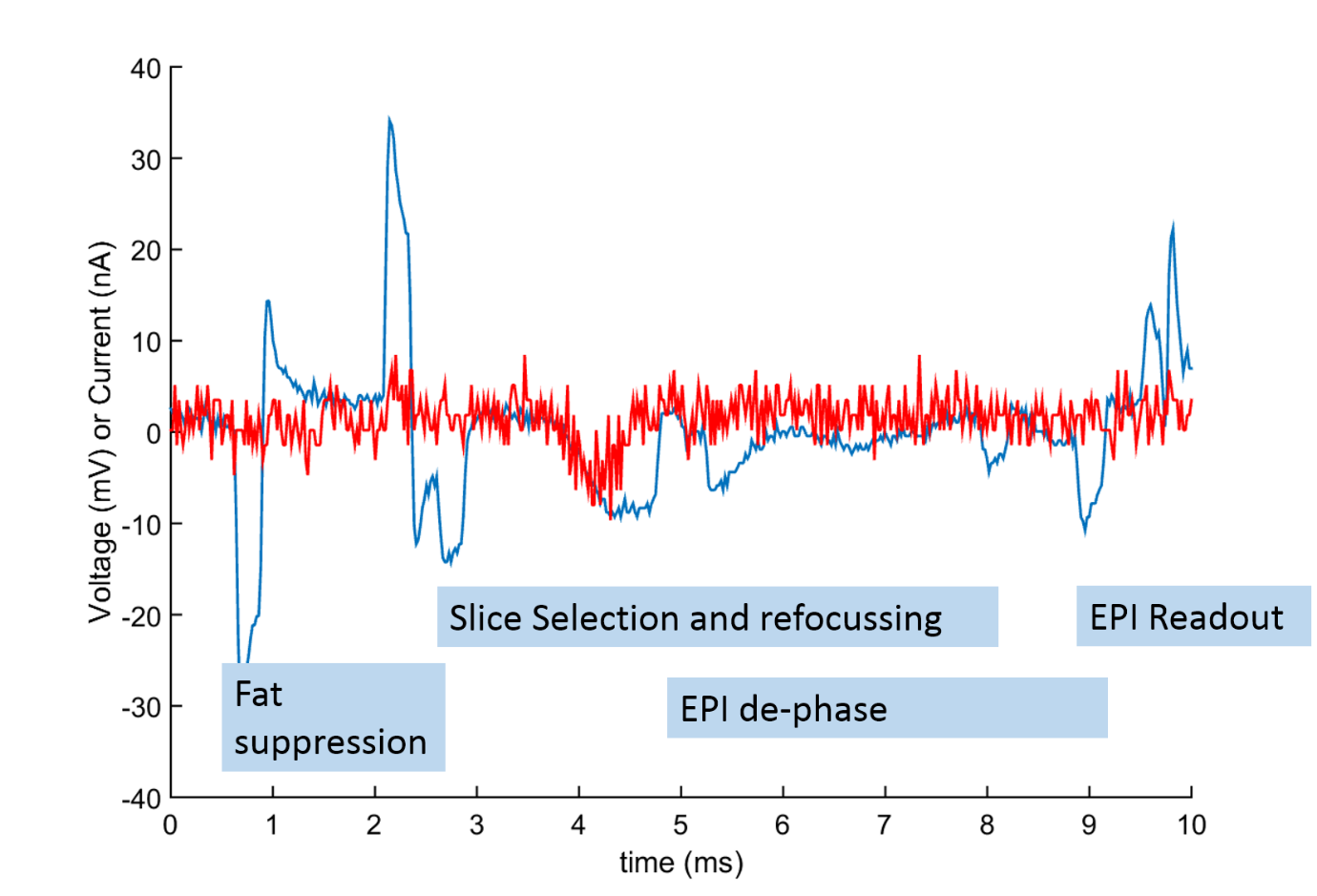


*Figure S.1: A 10 ms excerpt from a recording of the stimulus driver voltages and current during the excitation and pre-EPI readout part of the multiband fMRI sequence. Voltage output (blue trace with a mV scale) reaches a 35 mV peak during the fat-suppression crusher gradient pulses (all three gradients used) and is higher than during the echo-planar (EP) readout part which commences at 9 ms. During this excerpt the current (red with a nA scale) is maintained near zero with the RF pulse being the cause of the small current pulse at around 4 ms.*

## Radio-frequency Interactions

There are specific questions related to RF safety: does the presence of the INMS system in its entirety cause an increase in SAR in the trunk and wrist of the subject to exceed regulatory limits?; and more specifically, is there any likelihood of very localised heating of the tissue around the electrodes and the tip which is inserted into the nerve?

It is beyond the scope of this work to carry out detailed electromagnetic simulations at the resolution which would be needed since there would be many assumptions such as the head coil used, components within the scanner bore, body and electrode position. However, it is known that although there can be some propagation of the RF energy from the head coil down the bore and trunk, the RF levels will be two orders of magnitude lower below the shoulders than in the head. In addition, in common with good practice for the placement of ECG or similar leads, the INMS uses only a single point of contact and the subject is insulated (>2 cm) from any cables to avoid induction loops.

It is instructive therefore to consider just the 50 mm loop made by the wires going from electrodes to head-stage. Assuming a worst case of 1 µT RMS (only a factor of 10 reduction from head coil) at 300 MHz then the induced EMF would be 1.20 V. In a 50 Ω system this would be equivalent to 30 mW. As the fMRI sequence has an off-on pulse duty cycle of about 30:1 then this reduces the average power dissipated at this example level to around 1 mW average. If this power were dissipated local to the electrode then a high SAR could result of a few watts per kilogram. What is unknown is the potential for serious heating around the tip even at low average SAR.

In order to address this question, an experiment was conducted based on an egg albumen coagulation, essentially similar to that carried out by McGlone et al [2]. A test cell was constructed, as shown in Fig S.2 whereby the extent of local heating could be measured relative to power RF dissipated. A Photon Control (Burnaby, Canada) LNY2 fluorescent fibre-optic temperature probe was used to measure temperature variations during bench tests and during fMRI scans.


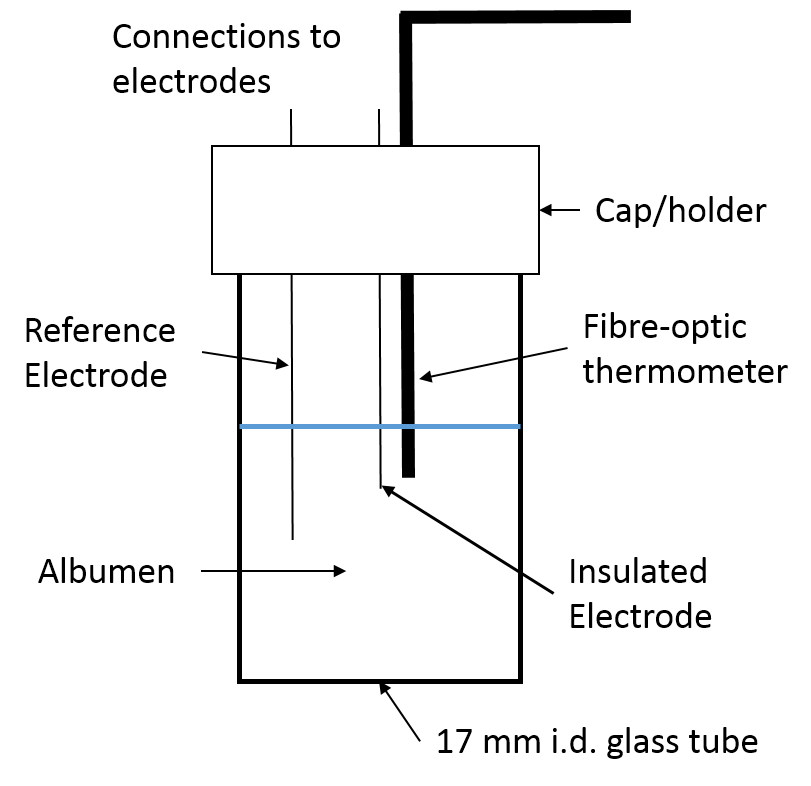


*Figure S.2 Test cell for RF heating experiments. The replaceable electrodes were held in a fixture such that the egg albumen covered 5 mm of the insulated (stimulus) electrode and 10 mm of the reference electrode. The 2 mm diameter fibre-optic thermometer was clamped such that its tip was 1.5 mm away from the electrode tip.*

Egg albumen reconstituted from food-quality dried egg white powder was used and mixed using supplier’s instructions (5 g powder to 21 g water). Albumen was added to the cell such that the stimulus electrode was covered by 5 mm of liquid. With a new stimulus electrode the low-frequency impedance of the test cell was of order 100 kΩ, on a par with that obtained using the bottle phantom or a subject and measured by the INMS as described in the paper. An RF connector was adapted to attach to the electrodes and an impedance of 88 – 104j Ω was obtained at 100 MHz, giving a return loss attributable to the loss in the albumen itself of -3.9 dB. Hence RF power could be dissipated in the albumen at a known level. A 1W power amplifier was available for bench testing and although this had a 100 MHz frequency limit, it is argued that at 300 MHz there would be very little difference in effect as it is the total and rate of energy absorbed which is important. The test cell is much smaller than the wavelength in both cases, so it is unlikely that the spatial extent of the current density would be dissimilar. Power was delivered to the cell, increasing in level and duration until coagulation was observed. As such we were able to relate the heating detected by the thermometer to observed coagulation levels. The results of typical tests are summarised in Table ST.1. These show that much greater than 100 mW continuous power delivered in excess of a few minutes is required for coagulation to take place. Coagulation is a severe outcome needing 80^o^C to occur, so this level of energy should in no way be seen as a limit. However, this experiment shows that local heating can occur even for low levels of power input, but the thermometer easily detects lower levels significantly below any potential problem. In our human study application both the body itself and the electrode wires will serve to conduct heat away. With the electrodes open and near the surface then our coagulation test is likely to be a worst case scenario.

| **Power (mW)** | **Temperature Rise (K)** | **Time (mins)** | **Energy (J)** | **Coagulation** |
| --- | --- | --- | --- | --- |
| 50 | 0.4 $\pm0.1$ | 10 | 30 | No |
| 100 | 1.0 $\pm0.1$ | 3 | 18 | No |
| 200 | 1.0 $\pm0.1$ | 1 | 12 | No |
| 400 | 2.0 $\pm0.1$ | 1 | 24 | No |
| 400 | 3.2 $\pm0.1$ | 2 | 77 | Possible |
| 500 | 5.0 $\pm0.1$ | 4 | 120 | Yes |
| 500 | No record (>5 K) | 10 | 300 | Substantial |

*Table ST.1: Table showing typical temperature rise for given dissipated power and observed coagulation on tip. Clearly rate of rise in temperature, as well as overall rise, is a good indicator of potential problems.*

The test cell was placed within the MR scanner and allowed to thermally stabilise. Initially, the cell was placed adjacent to, but not inside, the 7T Nova head-coil with a 50 mm diameter loop attached as an antenna. This antenna is the worst possible case scenario as usually the leads are connected to the head-stage inputs. The RF impedance of the head-stage inputs at 300 MHz was measured to be 17 – 200j Ω, even though 5 kΩ resistors are placed in-series, as is commonly used with MRI compatible EEG electrodes. The multiband fMRI experiment as described in the main text was run with a typical 1.8 W/kg SAR (57% of maximum) and 6.6 W average reported on scanner. Various orientations of the loop were arranged with no measurable heating (± 0.1 C).

The cell was then positioned at approximately the wrist position of a typical subject, the INMS system, head stage and electrode wires were attached. Again, no heating was observed (± 0.1 C) after 2 minutes scan time. This was repeated 6 times in quick succession and no temperature rise was ever observed.

## Initial human subject tests

As an attempt at the most realistic experiment possible, prior to conducting the first microstimulation experiment, a human subject was scanned with the following procedure: A slab of agar (4% with 0.5% salt by weight) 5mm thick and approximately 30 mm square was strapped to the wrist and electrical contact with the skin was ensured by using a smear of standard ECG electrode gel. Electrodes and the thermometer were inserted into the gel to replicate as closely as possible the geometry of the test cell described earlier. The whole wrist was thermally insulated. The INMS head-stage, cable routing and electrode wires were arranged as in the actual fMRI experiment. A full 4 minute fMRI 1.8 W/kg SAR sequence produced no evidence of heating (± 0.1 C) linked to scanner operation.

Having evidence that our temperature measurement would be able to indicate a potential problem well before any actual damage occuring, we are confident that there is no RF induced tissue heating around the electrode for this particular scanner, geometry and frequency. This will not necessarily be true for all scanners and operating frequencies. We would recommend therefore, for other applications, that the electrode connectors be fitted with non-magnetic 5 kΩ resistors in-series with the electrode. In light of our measurements and for our particular application (7T head scanning) this wasn’t thought to be necessary, plus it would make the electrode connectors very fragile.

# References

1. Reilly J.P. Peripheral-Nerve Stimulation By Induced Electric Currents - Exposure To Time-Varying Magnetic-Fields. *Medical & Biological Engineering & Computing* 27: 101-110, 1989.

2. McGlone, F., Kelly, E.F., Trulsson, M., Francis, S.T., Westling, G., Bowtell, R., 2002. Functional neuroimaging studies of human somatosensory cortex. *Behav. Brain Res*. 135, 147–58.
